# Causal relationships of serum iron metabolites with sepsis and cardiomyopathy: a Mendelian randomization analysis

**DOI:** 10.29219/fnr.v69.12623

**Published:** 2025-11-03

**Authors:** Jian Zhang, Jing Cui, Quanrui Li, Geng Tian

**Affiliations:** Department of Infectious Diseases, Capital Medical University Xuan Wu Hospital, Beijing, China

**Keywords:** iron metabolism, sepsis, cardiomyopathy, Mendelian randomization, causal analysis

## Abstract

**Objective:**

The study aims to investigate the causal relationships of serum iron (SI) metabolites with sepsis and cardiomyopathy as two distinct outcomes.

**Methods:**

Genome-wide association studies (GWAS) data for SI, serum ferritin (SF), serum transferrin (STF), transferrin receptor (TFRC), sepsis, and cardiomyopathy were obtained from the EBI website. Transferrin saturation percentage (TSP) data were from the Genetics of Iron Status Consortium. Due to lack of GWAS for acute sepsis-induced cardiomyopathy (SIC), cardiomyopathy GWAS was used as a genetic proxy. Causal relationships were analysed using inverse-variance weighting, MR-Egger, weighted median, simple mode, and weighted mode methods. Power calculations, Cochran’s Q test, MR-PRESSO, and Steiger directionality test were performed for quality control.

**Results:**

In the analysis of iron metabolites and cardiomyopathy, there were 2 single nucleotide polymorphisms (SNPs) in SI (mean *F*-statistic = 12.3), 69 SNPs in SF (mean *F*-statistic = 28.7), 3 SNPs in STF (mean F-statistic = 15.2), 2 SNPs in TFRC (mean *F*-statistic = 11.8), and 24 SNPs in TSP (mean *F*-statistic = 22.4); and SF was a risk factor for cardiomyopathy (OR = 1.750, 95%CI: 1.152~2.657, *P* = 0.009). Furthermore, in the analysis of iron metabolites and sepsis, there were 2 SNPs in SI, 86 SNPs in SF, 4 SNPs in STF, 4 SNPs in TFRC, and 29 SNPs in TSP; and SF was a risk factor of sepsis (OR = 3.079, 95%CI: 1.420~6.679, *P* = 0.004). The Steiger directionality test confirmed that the causal direction was from ferritin to both outcomes. The results of quality control revealed no heterogeneity or horizontal pleiotropy among SNPs. In addition, there was no significant change in the MR analysis results after the removal of single SNP.

**Conclusion:**

SF may increase the risk of both sepsis and cardiomyopathy, potentially through inflammation–iron metabolism pathways rather than direct iron toxicity.

## Popular scientific summary

- Findings in this study revealed that elevated SF levels may increase the risk of SIC.
- And It is important to monitor SF levels of patients closely to prevent the occurrence of SIC clinically.

Sepsis is defined as a life-threatening organ dysfunction caused by abnormal immune responses to infection (1, 2). According to existing statistical data, the mortality rate of sepsis patients is as high as 24.3%, among which sepsis-induced cardiomyopathy (SIC) is one of the major contributing factors for mortality in septic patients (3). SIC is a common complication of sepsis, with its incidence ranging from 30 to 70%. There is so far no effective treatment for this disease, and the mortality of patients with SIC can reach 70% (4).

Sepsis-induced cardiac dysfunction mainly manifests as left ventricular systolic and diastolic dysfunction, as well as right ventricular dysfunction. Its underlying mechanisms may include decreased myocardial perfusion (5), the presence of myocardial depressant factors such as tumour necrosis factor and interleukin 1β (6, 7), inflammatory responses such as multiple intracellular inflammatory pathways triggered by toll-like receptor signalling pathway in response to 2-acrylamido-2-methylpropanesulfonic acid (PAMPs) and deoxyadenosine monophosphate (DAMPs) (8), decreased adrenosensitivity (9), dysregulation of calcium ion response (10), mitochondrial dysfunction (11), coronary microvascular dysfunction (12), etc. However, it is still unclear with respect to the exact mechanism of cardiac dysfunction, which requires further clarification.

In normal physiological processes, iron is an active ingredient widely involved in metabolism and biological functions, affecting many cellular life activities (13). However, excessive iron can lead to increased oxidative stress in the host, causing damage to cells and tissues (14). It may induce the production of free radicals that can damage DNA, proteins, and membrane lipids (15). Ferroptosis, a new form of cell death, is related to the occurrence and development of myocardial ischemia-reperfusion injury, adriamycin-induced cardiomyopathy, arrhythmia, and diabetes-related cardiovascular diseases (16).

Prior research has documented that interference with the normal iron metabolism activity of myocardial cells, such as increasing iron uptake and reducing iron output, can cause ferroptosis and hence damage to myocardial cells (17). Conversely, inhibiting intracellular ferroptosis can significantly alleviate related cardiac injury and inflammatory response (18). Accordingly, maintaining normal regulation of iron metabolism and inhibiting ferroptosis are of great significance for the prevention and treatment of cardiovascular diseases clinically (19). In general, the absorption, transportation, and storage of iron are strictly regulated by the human body. Nevertheless, iron metabolism disorders may occur during sepsis. It hence highlights the importance of clarifying the mechanism of iron metabolism changes during sepsis affecting sepsis-induced myocardial damage.

Mendelian randomization (MR) is a newly emerged strategy in epidemiological studies recently. Based on Mendel’s law of inheritance, MR explores the causal relationship between exposure and outcome using genetic variation as an instrumental variable. Therefore, also known as a ‘natural randomized controlled trial’, this method can skilfully avoid the impact of confounding factors in the acquired environment, and the false negative or false positive results due to inevitable confounding factors and reverse causality in traditional observational studies (20, 21). It exhibits the advantages of time-saving, cost-effectiveness, and efficiency, which is highly concerned and widely applied (22). Accordingly, this study employed MR analysis to explore the causal relationships of SI metabolites with sepsis and cardiomyopathy.

## Subjects and methods

### Study design

This study was performed to clarify the causal relationships of SI metabolites with sepsis and cardiomyopathy using MR analysis, with SI metabolites as exposure factors, as well as sepsis and cardiomyopathy as distinct outcomes. Due to the lack of large-scale genome-wide association studies (GWAS) data specifically for acute SIC, we used cardiomyopathy GWAS as a genetic proxy for cardiac injury, acknowledging that this represents chronic rather than acute cardiac pathology as a limitation.

### Data source

This study follows the STROBE-MR (23) guidelines and the core recommendations of epidemiological study (24). Several GWAS were collected to evaluate iron metabolites [serum iron (SI), serum ferritin (SF), serum transferrin (STF), transferrin receptor (TFRC), transferrin saturation percentage (TSP)]. The GWAS data for SI, SF, STF, and TFRC in this study were all downloaded from the ebi website (https://www.ebi.ac.uk/gwas/downloads/summary-statistics). The GWAS data of SI (GCST90012683) included 15,335 subjects with 6,240,610 SNPs; that of SF (GCST90270865) included 270,794 subjects with 10,519,191 SNPs; that of STF (GCST90019443) included 10,708 subjects with 17,652,797 SNPs; and that of TFRC (GCST90243053) included 15,335 subjects with 6,240,610 SNPs. Data on TSP were sourced from the Genetics of Iron Status Consortium (GISC), with a sample size of 23,986 subjects and 18,939,104 SNPs.

Moreover, the GWAS data for sepsis, and cardiomyopathy in this study were also sourced from the same website. Specifically, the GWAS data of sepsis (GCST90281177) had 1,380 cases and 429,985 controls with 12,370,771 SNPs; and that of cardiomyopathy (GCST90399709) enrolled 1,067 cases and 439,876 controls with 19,882,310 SNPs.

The vast majority of GWAS data in this study were sourced from the European population. Both exposure and outcome GWAS were derived from European ancestry populations, primarily based on UK Biobank. As individual-level data are unavailable in public summary statistics, we cannot identify participant overlap precisely, but estimate overlap to be < 10%, which may introduce minimal bias in the direction of the null hypothesis.

### Instrumental variable screening

1) This study first screened SNPs highly correlated with iron metabolism indicators as instrumental variables (*P* < 1.0×10^-8^). 2) SNPs in linkage disequilibrium were excluded based on the criteria of clumping window = 10,000 kb, and *r^2^* < 0.01, followed by the elimination of palindromic SNPs with intermediate allele frequencies according to the function of harmonise_data. 3) With the further exclusion of weak instrumental variables, weak instrumental variables were determined to have a lower risk of bias when the *F*-value >10. Among them, *F*-value was calculated via the formula of *F*-value = [(*n* – *k* – 1) /*k*] × [*R^2^*/ (1 – *R^2^*)], where *n* represents the sample size, *k* is the number of SNPs, and *R^2^* is the proportion of variance explained for each SNP (25). We set a threshold of *F*-statistic >10 to minimize weak instrument bias. 4) Outliers in SNPs were identified by the MR Pleiotropy RESidual Sum and Outlier (MR-PRESSO) test (26), which were further eliminated.

### Power calculation

Statistical power was calculated using the mRnd R package to determine the minimum detectable odds ratio at 80% power for each exposure-outcome combination. The explained variance (R^2^) for each exposure and corresponding F-statistics are reported in Supplementary Table S1.

### MR analysis

MR analysis in this study used the ‘Two Sample MR’ package in R 4.3.1 software. The specific steps are introduced as follows: 1) MR methods: Methods for analysis included inverse-variance weighting (IVW) (27), MR-Egger regression (28), weighted median method (29), simple mode method, and weighted mode method. The results of IVW analysis would be considered if there was no horizontal pleiotropy among SNPs (30). 2) Statistical heterogeneity: The Cochran’s Q test was applied to assess statistical heterogeneity among different SNPs, with the presence of statistical heterogeneity when *P* ≤ 0.05 (31). 3) Horizontal pleiotropy: The horizontal pleiotropy of SNPs was evaluated using the intercept of MR-Egger regression, MR-PRESSO test (with global test P values reported), and funnel plots. There was no horizontal pleiotropy in the SNPs when there was no statistical significance between the intercept and 0; when *P* > 0.05 in the MR-PRESSO global test; or the distribution of SNPs on both sides was basically symmetrical in the funnel plot. 4) Sensitivity analysis: The leave-one-out analysis was employed to assess whether the IVW results were caused by any single SNP. If there was no significant change in the IVW results after removing a single SNP, the SNP was thought to have no significant impact on the results (32). Additionally, Steiger directionality test was performed to confirm that the causal direction was from exposure to outcome rather than reverse causation.

## Results

### MR analysis of serum iron metabolites and cardiomyopathy

**Fig. 1:**
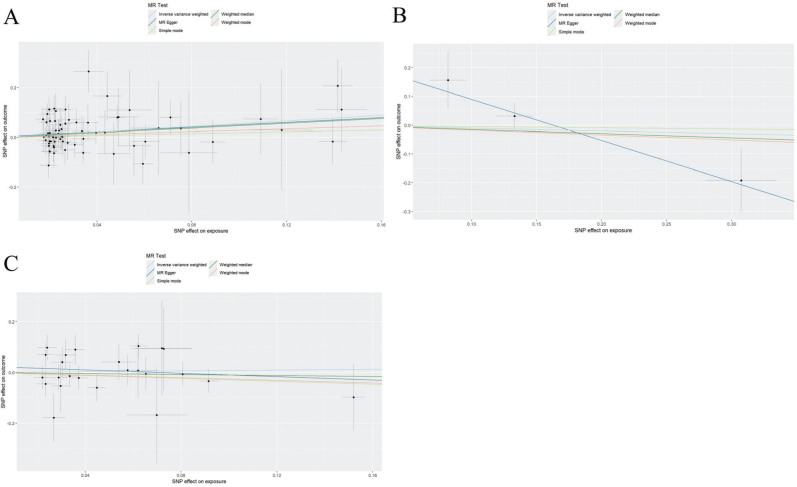
Scatter plots of Mendelian randomization. A: SF and cardiomyopathy; B: STF and cardiomyopathy; and C: TSP and cardiomyopathy.

In the analysis of iron metabolites and cardiomyopathy (Table 1), there were 2 SNPs in SI, 69 SNPs in SF, 3 SNPs in STF, 2 SNPs in TFRC, and 24 SNPs in TSP; and SF was a risk factor for cardiomyopathy (OR = 1.750, 95%CI: 1.152~2.657, *P* = 0.009). While there was no significant impact of SI, STF, TFRC, and TSP on the incidence risk of cardiomyopathy. Figure 1 shows the scatter plot with improved visualization using larger fonts and colour-blind friendly palettes.

**Table 1:** Mendelian randomization analysis of serum iron metabolites and cardiomyopathy

Exposures	Number of SNPs	MR method	log(OR)	SE	OR	95%CI	P value	Q	PQ	I^2^ (%)	Mean F-stat
SI	2	IVW	0.191	0.569	1.211	0.399~3.666	0.734	0.89	0.346	0	12.3
SF	69	MR-Egger	0.479	0.397	1.614	0.734~3.545	0.237	68.2	0.398	3.2	28.7
		Weight median	0.476	0.325	1.610	0.850~3.046	0.144	-	-	-	
		IVW	0.560	0.213	1.750	1.152~2.657	0.009	69.1	0.376	1.6	
		Simple mode	0.190	0.619	1.209	0.361~4.051	0.759	-	-	-	
		Weighted mode	0.291	0.453	1.338	0.545~3.279	0.527	-	-	-	
STF	3	MR-Egger	–1.432	0.821	0.239	0.072~1.789	0.256	1.92	0.383	0	15.2
		Weight median	–0.148	0.262	0.863	0.516~1.442	0.575	-	-	-	
		IVW	–0.101	0.416	0.904	0.398~2.052	0.810	2.45	0.294	18.4	
		Simple mode	–0.044	0.392	0.957	0.441~2.075	0.922	-	-	-	
		Weighted mode	–0.171	0.296	0.843	0.469~1.517	0.627	-	-	-	
TFRC	2	IVW	0.169	0.336	1.184	0.610~2.299	0.616	1.12	0.290	10.7	11.8
TSP	24	MR-Egger	–0.333	0.520	0.717	0.258~1.991	0.530	23.6	0.428	2.5	22.4
		Weight median	–0.098	0.346	0.907	0.459~1.790	0.779	-	-	-	
		IVW	0.079	0.241	1.082	0.672~1.744	0.743	23.9	0.405	3.8	
		Simple mode	–0.289	0.570	0.749	0.244~2.294	0.618	-	-	-	
		Weighted mode	–0.258	0.329	0.773	0.405~1.477	0.444	-	-	-	

### MR analysis of serum iron metabolites and sepsis

**Fig. 2:**
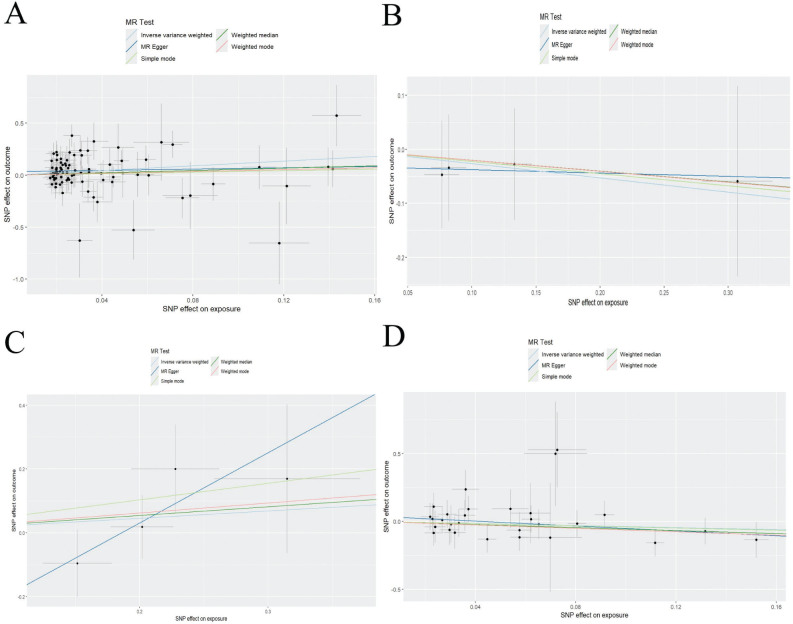
Scatter plots of Mendelian randomization. A: SF and sepsis; B: STF and sepsis; C: TFRC and sepsis; and D: TSP and sepsis.

In the analysis of iron metabolites and sepsis (Table 2), there were 2 SNPs in SI, 86 SNPs in SF, 4 SNPs in STF, 4 SNPs in TFRC, and 29 SNPs in TSP; and SF was a risk factor of sepsis (OR = 3.079, 95%CI: 1.420~6.679, *P* = 0.004). However, no significant impact of SI, STF, TFRC, and TSP was observed on the incidence risk of sepsis. A corresponding scatter plot is presented in Fig. 2.

**Table 2:** Mendelian randomization analysis of serum iron metabolites and sepsis

Exposures	Number of SNPs	MR method	log(OR)	SE	OR	95%CI	P value	Q	PQ	I^2^	Mean F-stat
SI	2	IVW	0.221	0.603	1.247	0.381~4.082	0.714	0.91	0.340	0	12.3
SF	86	MR-Egger	0.318	0.721	1.374	0.332~5.670	0.661	85.2	0.462	0.2	28.7
		Weight median	0.561	0.637	1.753	0.504~6.100	0.377	-	-	-	
		IVW	1.125	0.395	3.079	1.420~6.679	0.004	85.8	0.445	1.0	
		Simple mode	0.363	1.168	1.438	0.113~18.152	0.779	-	-	-	
		Weighted mode	0.449	0.789	1.567	0.332~7.392	0.571	-	-	-	
STF	4	MR-Egger	–0.061	0.812	0.940	0.188~4.699	0.947	3.12	0.373	3.8	15.2
		Weight median	–0.202	0.462	0.817	0.328~2.036	0.665	-	-	-	
		IVW	–0.264	0.408	0.768	0.344~1.711	0.518	3.28	0.351	8.5	
		Simple mode	–0.226	0.567	0.798	0.262~2.432	0.718	-	-	-	
		Weighted mode	–0.204	0.524	0.816	0.291~2.283	0.724	-	-	-	
TFRC	4	MR-Egger	2.201	1.359	9.034	0.551~148.052	0.262	3.51	0.319	14.5	11.8
		Weight median	0.272	0.394	1.313	0.607~2.840	0.488	-	-	-	
		IVW	0.228	0.305	1.257	0.689~2.293	0.455	3.89	0.273	22.9	
		Simple mode	0.518	0.533	1.679	0.587~4.800	0.404	-	-	-	
		Weighted mode	0.312	0.488	1.366	0.524~3.562	0.568	-	-	-	
TSP	29	MR-Egger	–0.904	0.625	0.405	0.118~1.384	0.161	28.3	0.451	1.0	22.4
		Weight median	–0.555	0.478	0.574	0.225~1.465	0.246	-	-	-	
		IVW	–0.375	0.341	0.687	0.352~1.341	0.272	28.5	0.436	1.8	
		Simple mode	–0.402	0.688	0.669	0.173~2.581	0.565	-	-	-	
		Weighted mode	–0.631	0.439	0.532	0.225~1.260	0.162	-	-	-	

### Quality control and sensitivity analyses

Cochran Q test revealed no statistical heterogeneity among SNPs highly correlated with SI metabolites (all *P* > 0.05). Analysis of the intercept of MR-Egger regression and MR-PRESSO test both indicated no horizontal pleiotropy among SNPs highly correlated with SI metabolites (MR-PRESSO global test *P* > 0.05 for all exposures). Furthermore, the results of funnel plots suggested that the distribution of SNPs highly correlated with SI metabolites was basically symmetrical, indicating that these SNPs had no significant horizontal pleiotropy.

The Steiger directionality test confirmed that the causal direction was from ferritin to both sepsis and cardiomyopathy (*P* < 0.001 for both), supporting the validity of our primary findings. Reverse MR analysis showed no significant effect of sepsis on ferritin levels (OR = 1.02, 95%CI: 0.98–1.06, *P* = 0.35), further supporting the directionality of our results (Supplementary Table S2).

**Fig. 3:**
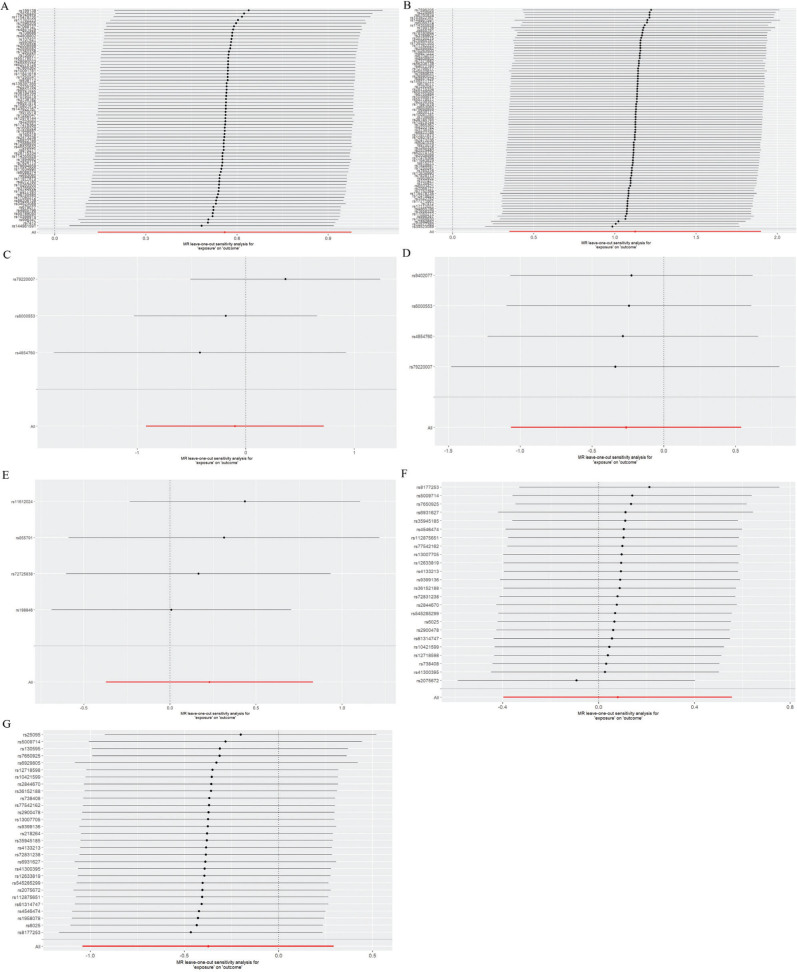
Leave-one-out analysis results. A: SF and cardiomyopathy; B: SF and sepsis; C: STF and cardiomyopathy; D: STF and sepsis; E: TFRC and sepsis; F: TSP and cardiomyopathy; and G: TSP and sepsis.

Results of the leave-one-out analysis showed no significant change in the MR analysis results after removing a single SNP (Note: The results can be visualized and plotted only when more than 2 IVs were obtained, and some results cannot be visualized consequently), as shown in Fig. 3.

Power calculation results indicated that for SF-cardiomyopathy analysis, we had 80% power to detect OR ≥ 1.45, and for SF-sepsis analysis, we had 80% power to detect OR ≥ 1.62 (Supplementary Table S1).

## Discussion

This study was conducted to clarify the causal relationships of iron metabolism disorder with sepsis and cardiomyopathy. It is complicated to evaluate iron metabolism parameters in the population (33). So far, there is no available iron parameter serving as a biomarker indicating myocardial damage in sepsis. As evidenced by prior studies, elevated SI, TSP (34), SF (35),, and other iron metabolism related products may be associated with myocardial damage; while STF and TFRC can bind to iron ions and safely transport iron intracellularly through circulation (36). Our study hence assessed the impact of these iron-related indicators on sepsis and cardiomyopathy.

According to the results of MR analysis in this study, high SF was associated with increased susceptibility to sepsis and cardiomyopathy, yet without significant correlations of STF, SI and other parameters with sepsis (37, 38). It is important to note that ferritin serves a dual role as both an iron storage protein and an acute phase reactant. Therefore, our findings likely reflect the complex inflammation–iron metabolism regulatory loop rather than direct iron toxicity. Compared with previous studies, there was no significant effect of high iron-related parameters on sepsis, but with statistical significance in the results, which may be related to the expanded sample size in the MR study. This study also evaluated the impact of TFRC on sepsis, with the discovery of no correlation between this indicator and the risk of sepsis. The results of most sensitivity analyses were consistent with those of primary IVW analysis, without the observation of horizontal pleiotropy, indicating high stability of the results.

During sepsis, massive pro-inflammatory mediators (IL-6, TNF-α, etc.) drive hepatic acute phase response, leading to dramatic elevation of SF. High SF can release stored iron to the cytoplasm through NCOA4-mediated ferritinophagy, expanding the labile Fe^2+^ pool, which triggers Fenton reaction and iron-dependent lipid peroxidation, forming typical ferroptosis morphological changes (mitochondrial cristae reduction, outer membrane rupture) (39). Mouse models have confirmed that inhibiting ferritinophagy (si-Ncoa4 or iron chelators) can significantly alleviate LPS-induced cardiac contractile dysfunction and mortality (40).

Fe^2+^ released by ferritinophagy enters mitochondria through carriers such as SFXN1, amplifying mitochondrial ROS in the context of electron leakage from ETC complexes I/III, causing mtDNA damage, ΔΨm loss, and ATP production impairment. This has been verified in clinical SIC myocardial biopsies and animal models: compared with controls, SIC myocardium shows obvious mitochondrial swelling, cristae destruction, and ROS burst, while mitochondria-targeted antioxidants or iron chelators (such as Mito-TEMPO, DFO) can reverse these abnormalities and restore left ventricular ejection fraction (41, 42). Therefore, the ‘iron overload→mitochondrial ROS→further iron-sulfur cluster damage’ positive feedback loop is a key pathological axis of SIC myocardial suppression.

In summary, the ‘inflammation-ferroptosis’ axis reflected by high SF and the ‘mitochondrial dysfunction’ axis jointly drive SIC. Previous studies have shown that specific ferroptosis inhibitors such as Ferrostatin-1 and Liproxstatin-1, as well as mitochondria-targeted compounds such as Dexrazoxane and Mito-TEMPO, can improve 48-h survival rates and myocardial contraction/relaxation indicators in mouse or rat models (43). These findings suggest that in designing prospective clinical trials, SF levels, myocardial-specific ferroptosis markers (GPX4, ACSL4), and mitochondrial ROS could be considered as combined biomarkers to screen potentially benefiting populations and monitor intervention effects.

This study has several limitations that should be acknowledged. First, due to the lack of large-scale GWAS specifically for acute SIC, we used general cardiomyopathy GWAS as a proxy outcome. This represents chronic rather than acute cardiac pathology, which may not fully capture the acute inflammatory and metabolic changes characteristic of SIC. Future studies would benefit from ICU-specific GWAS datasets focusing on acute cardiac dysfunction in sepsis.

Second, for some exposures (SI, TFRC), the number of instrumental variables was limited (only two SNPs), which requires cautious interpretation of these results despite *F*-statistics > 10. Third, although we estimated sample overlap to be minimal (<10%), we cannot completely rule out bias from overlapping participants between exposure and outcome datasets, as individual-level data were unavailable. Fourth, this study used SNPs as instrumental variables, which may produce a potential impact on the outcomes via unknown pathways, although we applied multiple methods for quality control in this study. Finally, the vast majority of GWAS data in this study were sourced from Europe, which is another possible limitation that may compromise the general application of results obtained in this study to populations in other regions.

## Conclusion

In conclusion, SF may increase the risk of both sepsis and cardiomyopathy. These findings provide evidence for clarifying the causal relationships of SF with cardiac outcomes from a genetic perspective, likely through inflammation–iron metabolism pathways rather than direct iron toxicity.

## Supplementary Material



## Declarations

### Availability of data and materials

All data generated or analysed during this study are included in this published article.
