# Supplementary material for: Causal relationships of serum iron metabolites with sepsis and cardiomyopathy: a Mendelian randomization analysis
Source: Food Nutr Res. 2025 Nov 3;69:10.29219/fnr.v69.12623. doi: 10.29219/fnr.v69.12623 (PMC12664298; doi:10.29219/fnr.v69.12623)
Supplement: Supplementary file 1 [file FNR-69-12623-s1.docx]

**Supplementary Materials**

**Supplementary Table S1. Power calculation results for Mendelian randomization analyses**

**A. Iron metabolites and cardiomyopathy**

| **Exposure** | **Number of SNPs** | **Total R² (%)** | **Mean F-statistic** | **Sample size (cases/controls)** | **Minimum detectable OR (80% power)** | **Actual OR (95% CI)** | **Power for observed OR (%)** |
| --- | --- | --- | --- | --- | --- | --- | --- |
| SI | 2 | 0.16 | 12.3 | 1,067/439,876 | 1.85 | 1.211 (0.399-3.666) | 28.4 |
| SF | 69 | 1.57 | 28.7 | 1,067/439,876 | 1.45 | 1.750 (1.152-2.657)* | 87.2 |
| STF | 3 | 0.28 | 15.2 | 1,067/439,876 | 1.72 | 0.904 (0.398-2.052) | NA† |
| TFRC | 2 | 0.15 | 11.8 | 1,067/439,876 | 1.88 | 1.184 (0.610-2.299) | 26.8 |
| TSP | 24 | 1.22 | 22.4 | 1,067/439,876 | 1.51 | 1.082 (0.672-1.744) | 23.5 |

**B. Iron metabolites and sepsis**

| **Exposure** | **Number of SNPs** | **Total R² (%)** | **Mean F-statistic** | **Sample size (cases/controls)** | **Minimum detectable OR (80% power)** | **Actual OR (95% CI)** | **Power for observed OR (%)** |
| --- | --- | --- | --- | --- | --- | --- | --- |
| SI | 2 | 0.16 | 12.3 | 1,380/429,985 | 1.78 | 1.247 (0.381-4.082) | 31.2 |
| SF | 86 | 1.96 | 28.7 | 1,380/429,985 | 1.62 | 3.079 (1.420-6.679)* | 92.8 |
| STF | 4 | 0.37 | 15.2 | 1,380/429,985 | 1.68 | 0.768 (0.344-1.711) | NA† |
| TFRC | 4 | 0.31 | 11.8 | 1,380/429,985 | 1.72 | 1.257 (0.689-2.293) | 33.7 |
| TSP | 29 | 1.48 | 22.4 | 1,380/429,985 | 1.44 | 0.687 (0.352-1.341) | NA† |

*Statistically significant (P < 0.05)
†Power calculation not applicable for protective effects (OR < 1)
R²: Proportion of variance in the exposure explained by the genetic instruments
F-statistic: Measure of instrument strength (F > 10 indicates strong instruments)
Power calculations performed using mRnd package in R (version 4.3.1)

**Supplementary Table S2. Directionality tests and reverse Mendelian randomization analyses**

**A. Steiger directionality test results**

| **Exposure-Outcome Pair** | **Steiger Z-statistic** | **P-value** | **Direction** | **Inference** |
| --- | --- | --- | --- | --- |
| SF → Cardiomyopathy | 8.24 | <0.001 | Correct | Causal direction confirmed |
| SF → Sepsis | 9.12 | <0.001 | Correct | Causal direction confirmed |
| STF → Cardiomyopathy | 4.56 | <0.001 | Correct | Causal direction confirmed |
| STF → Sepsis | 5.23 | <0.001 | Correct | Causal direction confirmed |
| TFRC → Cardiomyopathy | 3.89 | <0.001 | Correct | Causal direction confirmed |
| TFRC → Sepsis | 4.12 | <0.001 | Correct | Causal direction confirmed |
| TSP → Cardiomyopathy | 6.78 | <0.001 | Correct | Causal direction confirmed |
| TSP → Sepsis | 7.45 | <0.001 | Correct | Causal direction confirmed |

**B. Reverse Mendelian randomization analyses**

| **Analysis** | **Number of SNPs** | **Method** | **OR (95% CI)** | **P-value** | **Q-statistic** | **P_heterogeneity** | **I² (%)** |
| --- | --- | --- | --- | --- | --- | --- | --- |
| **Sepsis → SF** | 5 | IVW | 1.02 (0.98-1.06) | 0.35 | 4.23 | 0.38 | 5.4 |
|  |  | MR-Egger | 1.01 (0.96-1.07) | 0.68 | 4.21 | 0.31 | 28.7 |
|  |  | Weighted median | 1.03 (0.98-1.08) | 0.26 | - | - | - |
| **Sepsis → SI** | 5 | IVW | 0.99 (0.97-1.01) | 0.42 | 3.98 | 0.41 | 0.0 |
| **Sepsis → STF** | 5 | IVW | 1.01 (0.98-1.04) | 0.58 | 4.67 | 0.32 | 14.3 |
| **Sepsis → TFRC** | 5 | IVW | 0.98 (0.95-1.01) | 0.19 | 5.12 | 0.28 | 21.9 |
| **Sepsis → TSP** | 5 | IVW | 1.00 (0.97-1.03) | 0.92 | 3.45 | 0.49 | 0.0 |
| **Cardiomyopathy → SF** | 3 | IVW | 1.01 (0.97-1.05) | 0.67 | 2.89 | 0.24 | 30.8 |
|  |  | MR-Egger | 0.99 (0.91-1.08) | 0.89 | 2.78 | 0.10 | 64.0 |
|  |  | Weighted median | 1.00 (0.96-1.04) | 0.94 | - | - | - |
| **Cardiomyopathy → SI** | 3 | IVW | 0.98 (0.95-1.01) | 0.23 | 2.12 | 0.35 | 5.7 |
| **Cardiomyopathy → STF** | 3 | IVW | 1.00 (0.97-1.03) | 0.89 | 1.98 | 0.37 | 0.0 |
| **Cardiomyopathy → TFRC** | 3 | IVW | 0.99 (0.96-1.02) | 0.52 | 2.34 | 0.31 | 14.5 |
| **Cardiomyopathy → TSP** | 3 | IVW | 1.01 (0.98-1.04) | 0.48 | 2.67 | 0.26 | 25.1 |

IVW: Inverse-variance weighted; OR: Odds ratio; CI: Confidence interval
None of the reverse MR analyses showed significant causal effects (all P > 0.05), supporting the primary direction of causality from iron metabolites to disease outcomes.

**C. MR-Egger intercept test for pleiotropy in reverse analyses**

| **Analysis** | **Intercept** | **SE** | **P-value** | **Interpretation** |
| --- | --- | --- | --- | --- |
| Sepsis → SF | 0.002 | 0.008 | 0.82 | No evidence of pleiotropy |
| Cardiomyopathy → SF | 0.006 | 0.011 | 0.61 | No evidence of pleiotropy |
